# Vascular normalization: a strategy to recondition the tumor immune microenvironment

**DOI:** 10.1186/2051-1426-1-S1-P155

**Published:** 2013-11-07

**Authors:** Yuhui Huang, Jianping Yuan, Elda Righi, Dan G Duda, Dai Fukumura, Mark C Poznansky, Rakesh K Jain

**Affiliations:** 1 Massachusetts General Hospital, Boston, MA, USA

## 

**Figure 1:**
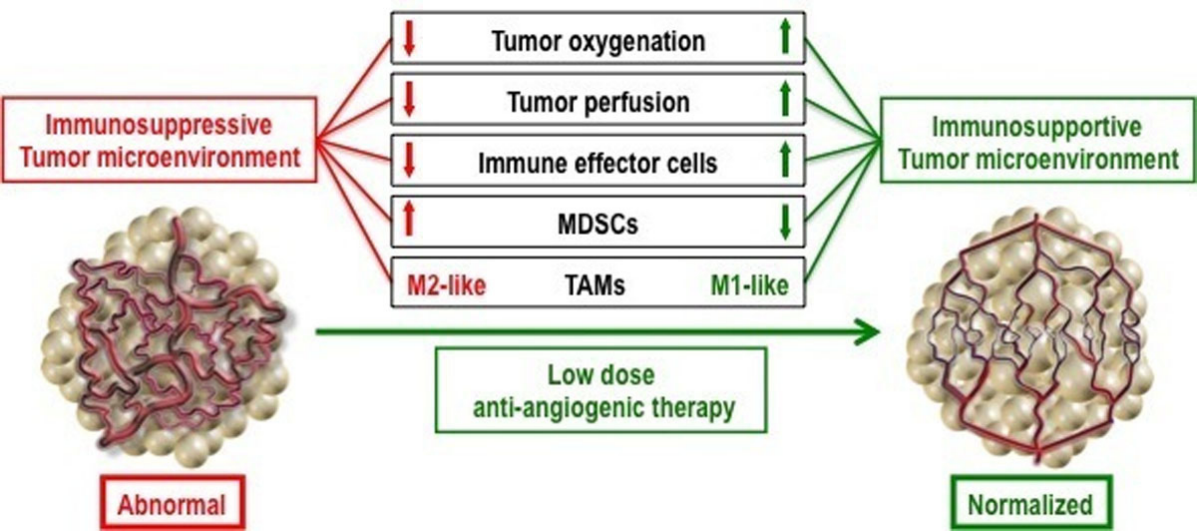
Lower “Vascular normalizing” dose of anti-angiogenic treatment reprograms the tumor microenvironment from immunosuppressive to immunosupportive.

Malignant tumors escape from host immune surveillance through multiple mechanisms. Of these, abnormal tumor vasculature and hypoxia are critical in establishing an immunosuppressive tumor microenvironment, and consequently, impeding an active cancer immunotherapy. Thus, we hypothesized that vascular normalization can recondition the tumor immune microenvironment and enhance a cancer immunotherapy. Here, we compared the dosage effects of anti-vascular endothelial growth factor receptor 2 antibody (DC101) treatments on tumor vasculature, and demonstrated that appropriate low dose DC101 treatment significantly improves functional tumor vessels and reduces tissue hypoxia, compared to control or high dose DC101 treatment in orthotopic preclinical breast tumor models. Consistently, our flow cytometry and gene profile analysis data showed that the lower doses are superior to the high doses at polarizing perivascular tumor-associated macrophages (TAMs) from M2-like to M1-like phenotype, in facilitating CD8+ T cell tumor infiltration, while reducing myeloid-derived suppressor cells (MDSCs) in the tumor parenchyma. Based on this mechanism, synchronizing vascular normalization with T cell activation induced by a whole cancer cell vaccine therapy significantly enhanced anti-cancer efficacy in a CD8+ T cell-dependent manner in both orthotopic and spontaneous breast cancer models. These findings indicate that vascular normalizing lower doses of anti-VEGFR2 antibody treatment can reprogram the tumor microenvironment away from immunosuppression toward potentiation of cancer vaccine therapies (Figure 1). Given that anti-angiogenic treatment is an established therapy in several solid cancers, our study suggests a potential translational strategy to improve the efficacy of cancer immunotherapies in clinic by combining with judicious low dose of anti-angiogenic treatments.

